# Needle Arthroscopy‐Guided Anatomic Reduction and Suture Button Fixation of the Distal Tibiofibular Syndesmosis

**DOI:** 10.1002/atn2.70332

**Published:** 2026-10-11

**Authors:** Błażej Grzegorz Wójtowicz, Bartosz Chlebanowski, Grzegorz Taborski, Grzegorz Kałużyński, Marcin Domżalski, Jedrzej Lesman

**Affiliations:** ^1^ Department of Orthopedics and Trauma Medical University of Lodz, University Hospital of Lodz No 2 Lodz Poland

## Abstract

Syndesmotic malreduction remains a major contributor to poor outcomes after ankle injuries, yet conventional fixation methods rely primarily on fluoroscopic assessment and may not fully reveal rotational or subtle translational malalignment. Needle arthroscopy offers direct visualization of the distal tibiofibular interval, enabling precise assessment of the injury and real‐time confirmation of reduction. This surgical technique describes a fully arthroscopic approach to anatomic reduction and dynamic stabilization of the syndesmosis using a suture button device. After arthroscopic identification of the pathological widening, a four‐cortex trans‐syndesmotic tunnel is created under fluoroscopic control, the medial button is deployed, and final reduction is achieved through symmetric tensioning while monitoring the syndesmosis with needle arthroscopy. This method allows accurate restoration of the fibular position within the incisura with minimal soft‐tissue disruption and may reduce the risk of malreduction associated with traditional techniques.

## Graphical abstract

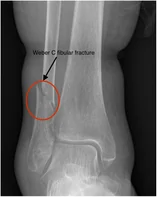

**VIDEO 1:** Step‐by‐step demonstration of needle arthroscopy‐assisted anatomic reduction and dynamic suture button fixation of an acute distal tibiofibular syndesmotic injury in a right ankle with the patient positioned supine on a radiolucent operating table. The video begins with the establishment of standard anteromedial and anterolateral portals and the introduction of a 1.9‐mm needle arthroscope for systematic evaluation of the anterior ankle compartment. Intra‐articular visualization shows pathologic widening of the distal tibiofibular syndesmosis, assessment of fibular instability using an arthroscopic probe, and identification of associated soft‐tissue pathology. Under simultaneous fluoroscopic guidance, a guidewire is inserted from the distal fibula into the tibia parallel to the ankle joint line, followed by the creation of a four‐cortex trans‐syndesmotic tunnel. A dynamic suture button construct is then introduced and deployed, with final reduction achieved through gradual symmetric tensioning under direct needle arthroscopic visualization. The video concludes with fluoroscopic confirmation of anatomic syndesmotic reduction, restoration of fibular position within the incisura, and final implant positioning. Video content can be viewed at https://doi.org/10.1002/atn2.70332.

Injuries to the distal tibiofibular syndesmosis represent a clinically significant source of ankle instability, particularly among physically active patients.^1^ Failure to achieve anatomic reduction in the acute setting has been shown to alter tibiotalar contact mechanics and predispose to persistent pain, functional impairment, and early degenerative joint disease. Conventional stabilization methods, most commonly trans‐syndesmotic screw fixation or dynamic suture button constructs, rely primarily on fluoroscopic assessment of reduction.^2^, ^3^ Although widely used, these approaches do not permit direct visualization of the syndesmotic interval and may therefore be associated with malreduction rates reported in the literature.^4^, ^5^

Needle arthroscopy has recently emerged as a minimally invasive modality that enables high‐resolution assessment of intra‐articular and periarticular structures while minimizing soft‐tissue disruption. Its small caliber allows direct access to the tibiofibular space, providing an unprecedented ability to evaluate the integrity of the syndesmotic ligaments and to confirm reduction dynamically during fixation.^6^

In this surgical technique, we describe a needle arthroscopic method for anatomic reduction and dynamic stabilization of the distal tibiofibular syndesmosis using needle arthroscopy in combination with a suture button device. This approach allows real‐time visualization of the reduction maneuver, objective confirmation of joint congruence, and precise control of final fixation, potentially improving accuracy while preserving the minimally invasive nature of the procedure.^7^, ^8^

## SURGICAL TECHNIQUE

The instructional video of syndesmosis repair is presented in Video 1.

### Patients Positioning and Preparation

The patient is placed supine on a radiolucent table with the foot positioned freely beyond the edge to allow full manipulation of the ankle. A tourniquet is not used. Syndesmotic stabilization is performed after definitive fixation of the associated malleolar fractures, with fluoroscopy confirming restoration of overall ankle alignment (Figures 1 and 2).

**FIGURE 1:**
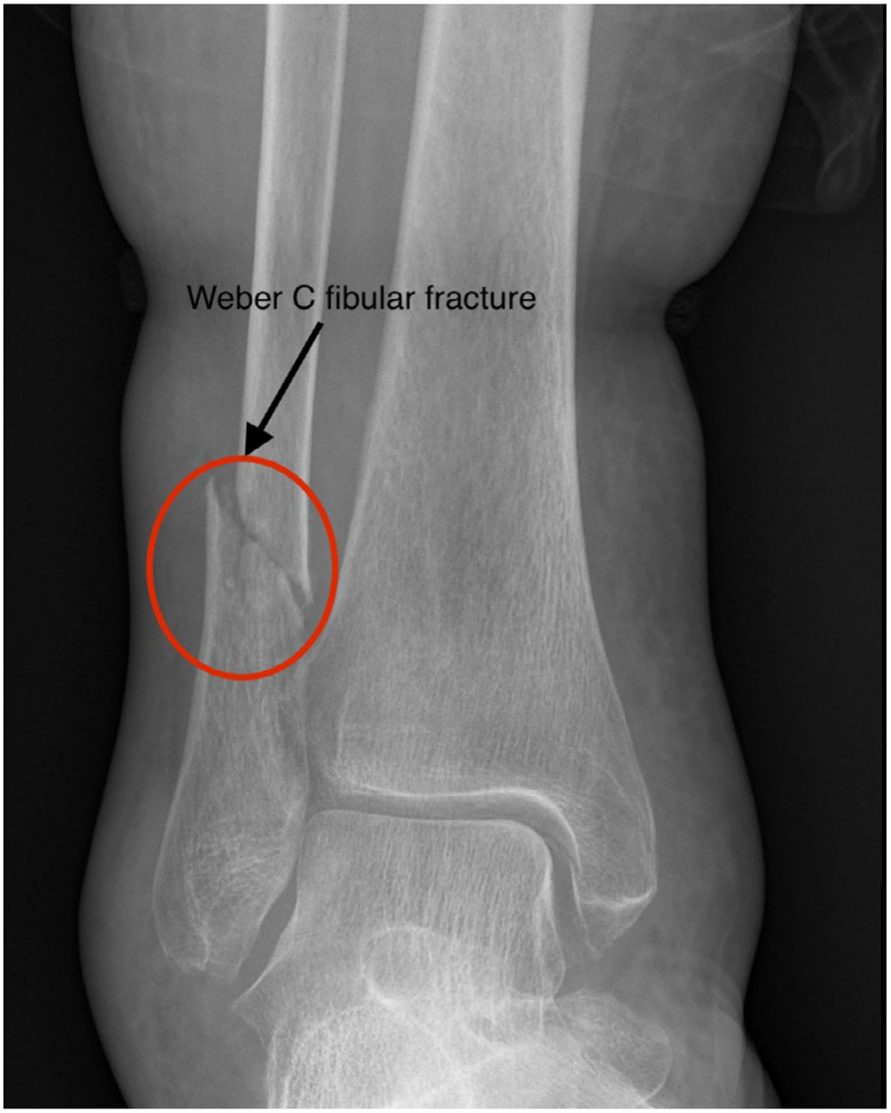
Preoperative anteroposterior radiograph of the right ankle, showing a Weber C fibular fracture (arrow), prior to surgical fixation.

**FIGURE 2:**
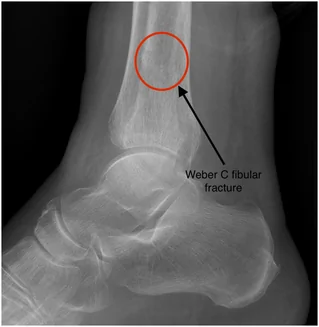
Preoperative lateral radiograph of the right ankle, showing the fibular fracture (arrow) and associated injury pattern before definitive fixation and syndesmotic stabilization.

After standard preparation and draping, the ankle is gently taken through a range of motion to assess residual instability. The limb is then positioned neutrally to facilitate creation of the anteromedial and anterolateral portals and subsequent needle arthroscopic evaluation.

### Equipment

The procedure requires a needle arthroscope (NanoScope; Arthrex, Naples, FL) with a standard fluid management system, as well as a blunt arthroscopic probe, a shaver, and small graspers; standard or nanoscale may be used when removal of loose bodies or minimal debridement is necessary. All implants and instrumentation required for dynamic suture button fixation should be prepared in advance.

### Needle Arthroscopy

Two standard anterior portals are used. The anteromedial portal is established first at the level of the ankle joint line, just medial to the tibialis anterior tendon. After a small skin incision, a blunt instrument is used to open the soft tissues, and the needle arthroscope is introduced into the anterior recess of the ankle. Continuous irrigation is initiated to maintain a clear field (Video 1).

A systematic arthroscopy is performed to evaluate the tibiotalar articular surfaces, anterior joint recess, and any synovial or chondral abnormalities (Figure 3). The medial and lateral gutters are inspected for loose bodies or soft‐tissue interposition. When necessary, loose fragments may be removed using nanoscale graspers or a small shaver introduced percutaneously.

**FIGURE 3:**
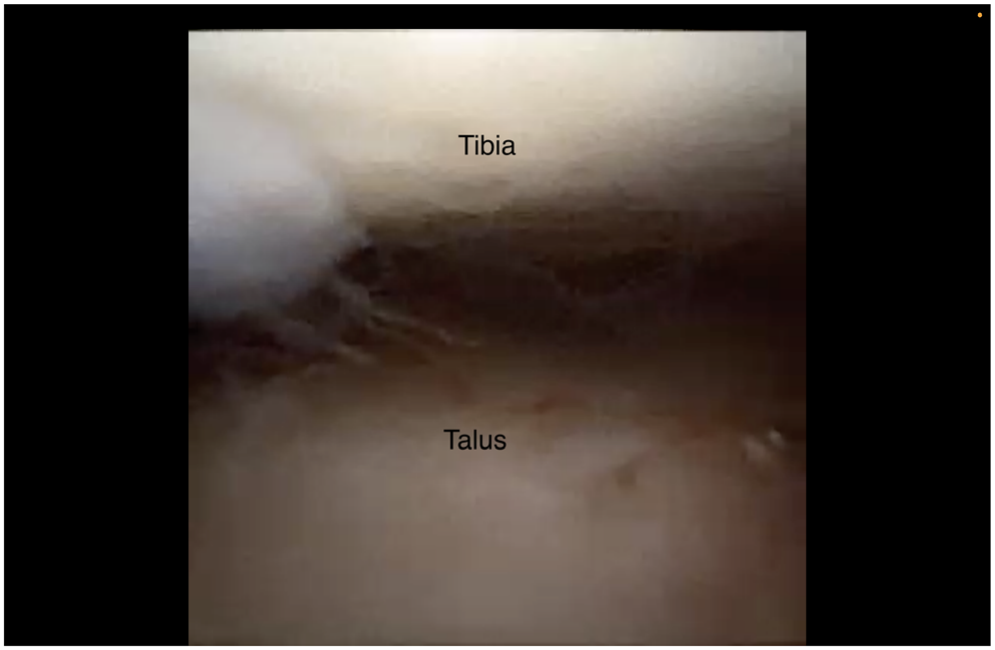
Needle arthroscopic view of the central anterior ankle compartment obtained through the anteromedial portal in a right ankle with the patient positioned supine. Visualization of the anterior gutter and tibiotalar articulation allows assessment of the articular cartilage, synovium, and associated intra‐articular pathology prior to syndesmotic fixation.

Under direct visualization from the anteromedial portal, an anterolateral portal is created. A needle is first inserted to confirm an appropriate trajectory toward the lateral gutter without risk to neurovascular structures. After confirming the position, a small incision is made, and a cannula or working tract is established (Figure 4). Switching the needle arthroscope to the anterolateral side allows a wider and more favorable viewing angle toward the distal tibiofibular articulation.

**FIGURE 4:**
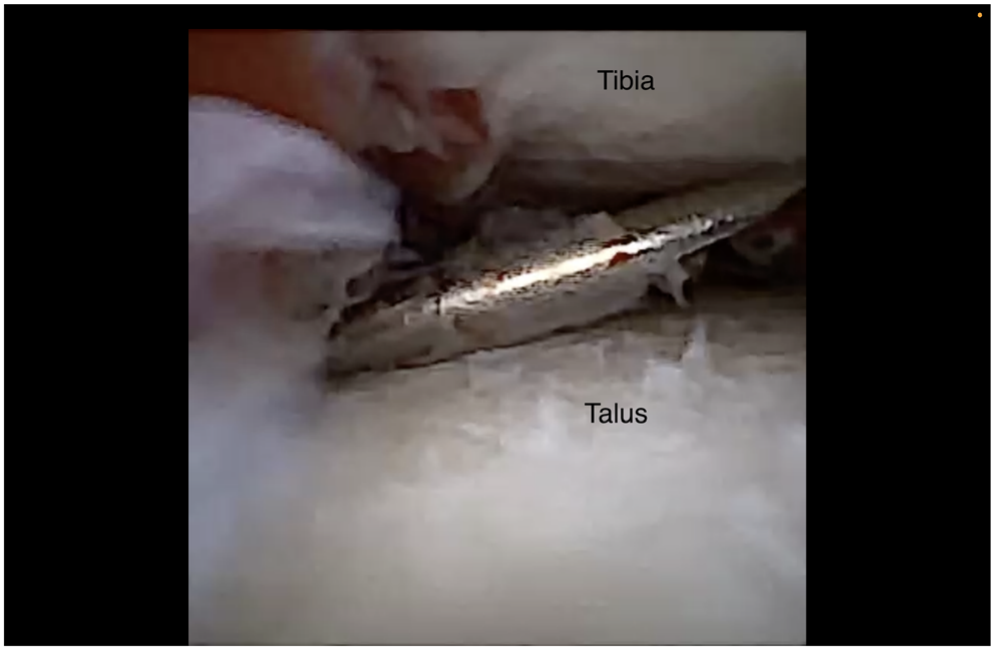
Needle arthroscopic view from the anteromedial portal showing the establishment of the anterolateral portal under direct visualization in a right ankle positioned supine. A spinal needle is introduced into the lateral gutter to confirm the safe trajectory and optimal location for subsequent anterolateral portal placement while minimizing the risk of iatrogenic injury.

With the arthroscope positioned laterally, the syndesmotic interval is directly visualized. The space between the distal fibula and tibia is assessed for pathologic widening, malalignment, or interposed tissue (Figure 5). A probe introduced through the opposite portal is used to evaluate the stability of the fibula relative to the tibia, assessing for excessive lateral translation or abnormal gapping during gentle stress maneuvers. This direct visualization confirms both the presence and extent of syndesmotic injury, guiding the subsequent reduction and fixation steps.

**FIGURE 5:**
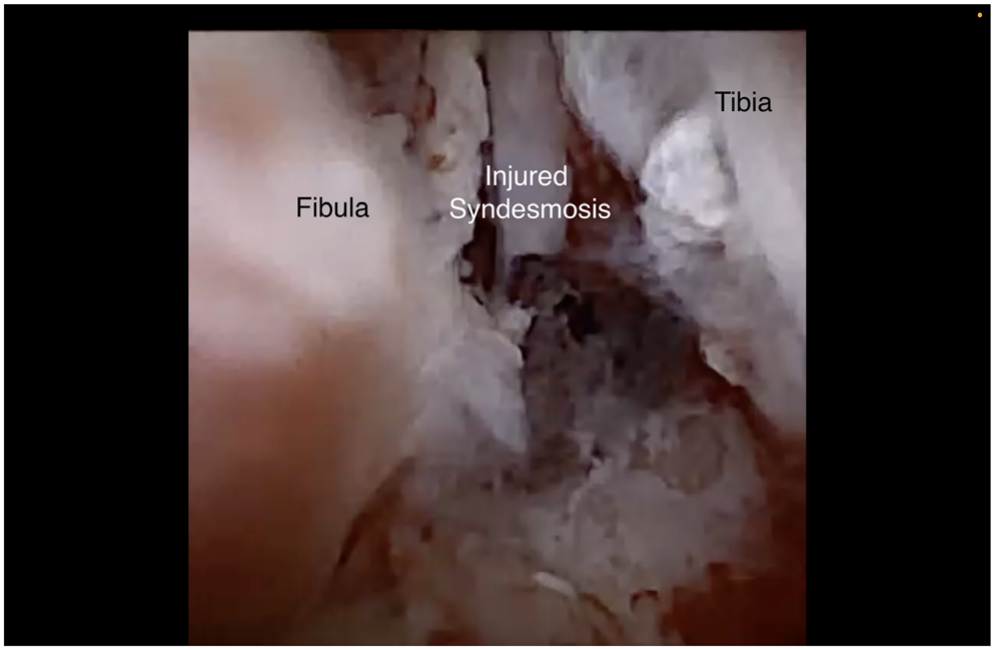
Needle arthroscopic view of the distal tibiofibular syndesmosis obtained through the anterolateral portal in a right ankle positioned supine. Pathologic widening and instability of the syndesmotic interval between the distal tibia and fibula are visible before reduction, confirming disruption of the distal tibiofibular syndesmosis.

Needle arthroscopy thus provides a minimally invasive yet precise assessment of the ankle joint and syndesmosis, enabling verification of both the pathology and the intraoperative correction.^9^

### Syndesmotic Reduction and Suture Button Fixation

With the needle arthroscope (NanoScope; Arthrex) positioned in the anterolateral portal, the distal tibiofibular interval is examined in detail. The widened syndesmotic space, the degree of malalignment, and any persistent separation between the tibia and fibula are assessed directly. This visualization provides an accurate understanding of the injury pattern and serves as the basis for guiding the reduction maneuvers that follow.

Once the pathology is clearly identified, attention is turned to creating the trans‐syndesmotic tunnel. Under fluoroscopic guidance, a guidewire is inserted percutaneously through the distal fibula toward the tibia. The trajectory is planned so that the wire advances parallel to the ankle joint line, ensuring that the tunnel is oriented appropriately relative to the syndesmotic plane. The correct positioning of the guidewire is verified in both anteroposterior and lateral fluoroscopic views. After confirming satisfactory alignment, a cannulated drill (dedicated 3.8‐mm drill; Arthrex) corresponding to the diameter recommended for the selected suture button system is advanced over the guidewire. A four‐cortex tunnel is created, traversing both fibular cortices and the medial and lateral tibial cortices. Care is taken to maintain the planned trajectory throughout drilling to avoid an eccentric placement or cortical breach.

After preparation of the osseous tunnel, the suture button (TightRope XP; Arthrex) construct is introduced. The medial button is deployed against the outer tibial cortex, and its seating is confirmed fluoroscopically. Proper apposition of the button to the cortical surface is essential to ensure stable fixation and to prevent soft‐tissue interposition. Once the medial button is fully seated, the guidewire is removed, and the sutures are retrieved through the lateral incision.

Reduction of the syndesmosis is achieved by gradual, alternating tensioning of the suture limbs. Throughout this maneuver, needle arthroscopy provides real‐time visualization of the tibiofibular interval, allowing the surgeon to directly observe the fibula translating and rotating back into its anatomic position within the incisura. The reduction is adjusted as necessary to correct both widening and rotational displacement. Tensioning is continued until the syndesmotic space appears anatomically restored arthroscopically, with no residual gapping or malalignment. A confirmatory fluoroscopic assessment is then performed to verify appropriate reduction and implant positioning (Figure 6).

**FIGURE 6:**
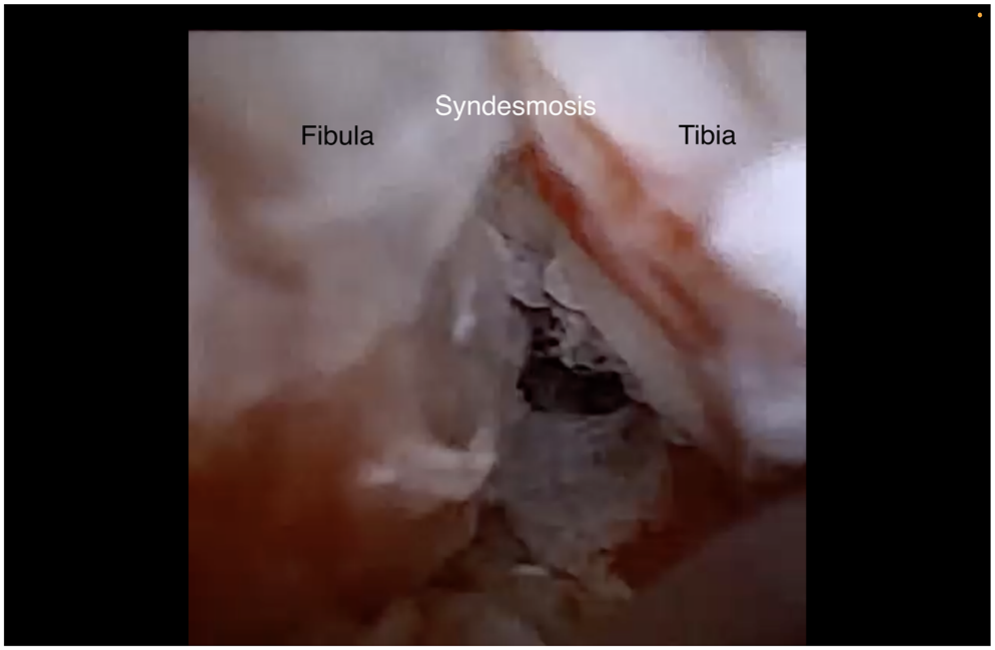
Needle arthroscopic view from the anterolateral portal showing anatomic reduction of the distal tibiofibular syndesmosis after stabilization with a dynamic suture button construct (TightRope; Arthrex, Naples, FL). Restoration of the fibular position within the incisura and elimination of syndesmotic widening are confirmed under direct visualization.

Once the desired reduction is achieved, the sutures are secured according to the manufacturer's recommendations, and any excess suture is trimmed. The portals are irrigated and closed in a routine fashion, completing the fixation stage of the procedure (Figures 7 and 8). The steps of the technique are listed in Table 1.

**FIGURE 7:**
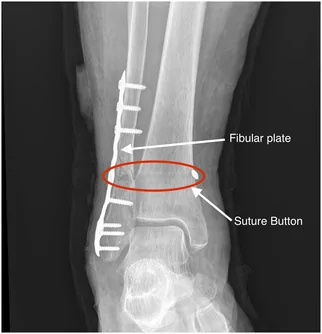
Postoperative anteroposterior radiograph of the right ankle obtained with the patient in the supine position after open reduction and internal fixation of the fibular fracture using a dedicated lateral fibular plate (Arthrex) with locking screws and dynamic syndesmotic stabilization using a TightRope suture button device (Arthrex). The radiograph shows restoration of fibular length, alignment, and syndesmotic congruency.

**FIGURE 8:**
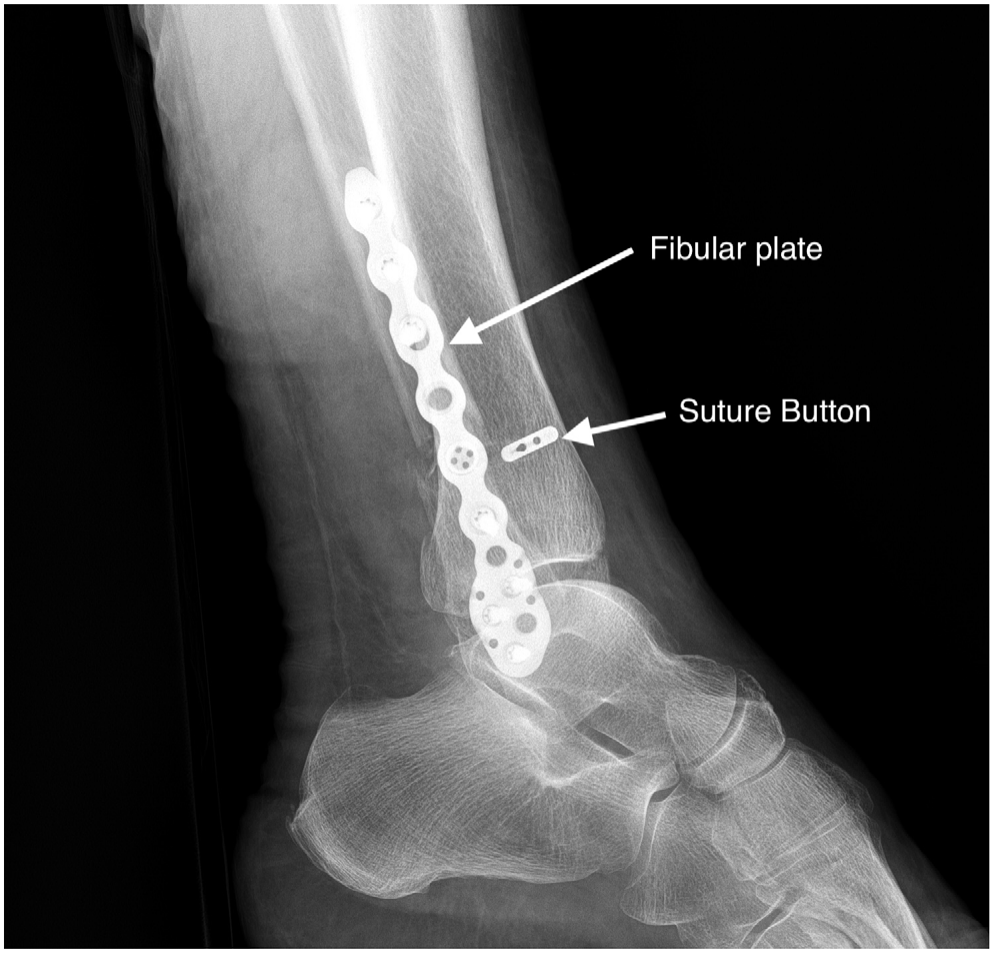
Postoperative lateral radiograph of the right ankle showing fixation of the fibular fracture with a dedicated lateral fibular plate and locking screws (Arthrex) in combination with dynamic syndesmotic stabilization using a TightRope suture button construct (Arthrex). Final implant positioning and restoration of ankle alignment are confirmed.

**TABLE 1:** Step‐by‐Step Description of the Technique

**Step**	**Description**
1	Patient positioning supine on a radiolucent table; syndesmotic fixation planned after definitive malleolar fracture fixation
2	Standard preparation and draping; fluoroscopic confirmation of overall ankle alignment
3	Establish an anteromedial portal at the ankle joint line and introduce the needle arthroscope
4	Perform needle arthroscopy of the anterior ankle compartment, assessing cartilage and gutters
5	Remove loose bodies or intra‐articular debris if present using small graspers and/or a shaver as needed
6	Create the anterolateral portal under direct visualization using a needle to confirm safe trajectory
7	Switch the needle arthroscope to the anterolateral portal to optimize visualization of the distal tibiofibular interval
8	Visualize and confirm syndesmotic injury and pathologic widening under needle arthroscopy
9	Insert a guidewire percutaneously from fibula to tibia under fluoroscopy with a trajectory parallel to the ankle joint line
10	Drill a four‐cortex trans‐syndesmotic tunnel using a cannulated drill matched to the selected suture button system
11	Pass the suture button construct through the tunnel and deploy the medial button against the outer tibial cortex under fluoroscopy
12	Remove the guidewire and retrieve the lateral sutures
13	Reduce the syndesmosis by gradual, symmetric tensioning while monitoring real‐time reduction with needle arthroscopy
14	Confirm final reduction and implant position with fluoroscopy
15	Secure the sutures, irrigate, and close portals in a routine fashion

## DISCUSSION

Achieving and maintaining anatomic reduction of the distal tibiofibular syndesmosis is essential for restoring normal ankle biomechanics.^10^ Even subtle malreduction has been shown to alter tibiotalar contact pressures, predisposing patients to persistent pain, diminished functional performance, and accelerated development of degenerative joint changes.^11^ ^,^ ^12^ Despite this, traditional fixation methods, whether trans‐syndesmotic screws or dynamic suture button constructs, are typically performed under fluoroscopic control alone. Fluoroscopy, although indispensable, provides only indirect information about rotational alignment and often fails to detect small degrees of diastasis.^13^ This limitation likely contributes to the clinically significant malreduction rates reported across multiple studies.^4^

The use of needle arthroscopy in acute syndesmotic injuries addresses this gap by enabling direct visualization of the tibiofibular interval. It is very small diameter allows atraumatic access to the joint with minimal soft‐tissue disruption while still providing high‐quality images sufficient to assess the width of the syndesmosis, the position of the fibula within the incisura, and the elimination of any interposed tissue.^14^ Unlike standard arthroscopy, needle arthroscopy offers these advantages with substantially lower morbidity and without the need for joint distraction, making it particularly suitable in cases where the primary goal is targeted visualization rather than extensive intra‐articular work.^15^

Incorporating needle arthroscopy into the fixation process also enhances control during reduction. The suture button construct permits gradual, symmetric tensioning, and the surgeon can monitor the corresponding movement of the fibula in real time. This dynamic feedback enables correction of both translational and rotational malalignment, which may otherwise go unrecognized when relying solely on fluoroscopic assessment.^16^ The technique described here therefore offers a more precise and reproducible method of restoring the native anatomy of the distal tibiofibular joint.

Additional benefits of this approach include the minimally invasive nature of the procedure, preservation of soft tissues, and elimination of the need for hardware removal, which remains a disadvantage of traditional screw fixation. The ability to combine fluoroscopic and arthroscopic assessment provides a dual‐modality confirmation of reduction, enhancing confidence in the final construct. Moreover, the technique can be performed efficiently in the same operative session after fixation of associated malleolar fractures without requiring patient repositioning or additional incisions.

However, several considerations should be acknowledged. The technique requires familiarity with needle arthroscopic instrumentation and an understanding of the arthroscopic anatomy of the anterior ankle. The quality of visualization may be influenced by intra‐articular bleeding or synovial proliferation, although in acute injuries these issues are generally limited. Finally, although the biomechanical principles supporting dynamic fixation are well established, further comparative clinical studies will be necessary to determine whether the addition of needle arthroscopy translates into improved long‐term functional outcomes or lower malreduction rates.^17^ Pearls, pitfalls, and risks of the technique are presented in Table 2.

**TABLE 2:** Pearls and Pitfalls

**Pearls**	**Pitfalls**
Direct visualization of the distal tibiofibular interval using needle arthroscopy allows real‐time confirmation of anatomic reduction	Reliance on fluoroscopy alone may fail to detect subtle rotational or translational malreduction
Systematic inspection of the anterior ankle compartment enables assessment of articular cartilage and identification of chondral lesions	Limited working space may restrict the treatment of extensive chondral pathology
Detection and removal of loose bodies or intra‐articular debris can be performed during the same procedure	Inadequate instrumentation may hinder removal of larger loose bodies
Drilling parallel to the ankle joint line helps reproduce native syndesmotic orientation	Oblique drilling may result in nonanatomic fixation or cortical breach
Four‐cortex tunnel creation provides secure seating of the suture button construct	Insufficient tunnel length may compromise medial button deployment
Gradual, symmetric tensioning under arthroscopic control facilitates controlled reduction	Excessive or asymmetric tensioning may lead to overcompression of the syndesmosis
Combined arthroscopic and fluoroscopic assessment improves confidence in final reduction	Familiarity with needle arthroscopy is required to avoid prolonged operative time

Despite these considerations, the described approach provides a reliable, reproducible, and minimally invasive method for achieving precise syndesmotic reduction. The combination of direct arthroscopic assessment with dynamic fixation offers substantial theoretical and practical advantages and represents a valuable addition to the evolving armamentarium for managing acute syndesmotic injuries. The advantages and disadvantages are listed in Table 3.

**TABLE 3:** Advantages and Disadvantages

**Advantages**	**Disadvantages**
Direct visualization of syndesmotic reduction	Requires needle arthroscopy experience
Detection of interposed tissue	Additional equipment required
Real‐time reduction control	Learning curve
Minimally invasive	Limited working space
Dynamic assessment of fibular position	Visualization may be impaired by bleeding

## DISCLOSURES

The authors (B.G.W., B.C., G.T., G.K., M.D., J.L.) declare that they have no known competing financial interests or personal relationships that could have appeared to influence the work reported in this article.

## References

[1] van Dijk CN , Longo UG , Loppini M , et al. Conservative and surgical management of acute isolated syndesmotic injuries: ESSKA‐AFAS consensus and guidelines. Knee Surg Sports Traumatol Arthrosc. 2016;24:1217‐1227. 10.1007/s00167-016-4017-1 26846658

[2] Takao M , Ochi M , Oae K , Naito K , Uchio Y . Diagnosis of a tear of the tibiofibular syndesmosis: The role of arthroscopy of the ankle. J Bone Joint Surg Br. 2003;85:324‐329. 10.1302/0301-620x.85b3.13174 12729102

[3] Stornebrink T , Walinga AB , Stufkens SAS , Kerkhoffs GMMJ . Wide‐awake needle arthroscopy of the anterior ankle: A standardized approach. Arthrosc Tech. 2024;13:102901. 10.1016/j.eats.2023.102901 PMC11056615 38690356

[4] Sagi HC , Shah AR , Sanders RW . The functional consequence of syndesmotic joint malreduction at a minimum 2‐year follow‐up. J Orthop Trauma. 2012;26:439‐443. 10.1097/BOT.0b013e31822a526a 22357084

[5] Miller AN , Carroll EA , Parker RJ , Boraiah S , Helfet DL , Lorich DG . Direct visualization for syndesmotic stabilization of ankle fractures. Foot Ankle Int. 2009;30:419‐426. 10.3113/FAI-2009-0419 19439142

[6] Colasanti CA , Mercer NP , Garcia JV , Kerkhoffs GMMJ , Kennedy JG . In‐office needle arthroscopy for the treatment of anterior ankle impingement yields high patient satisfaction with high rates of return to work and sport. Arthroscopy. 2022;38:1302‐1311. 10.1016/j.arthro.2021.09.016 34571184

[7] Duenes ML , Azam MT , Butler JJ , Weiss MB , Kennedy JG . In‐office needle arthroscopy for the foot and ankle. Arthroscopy. 2023;39:1129‐1130. 10.1016/j.arthro.2023.01.005 37019528

[8] Mioduszewski A , Wróbel M , Sroczyński J , Kłos G , Bocheński W , Nawrocki M . Stabilization of anterior aspect of distal tibiofibular syndesmosis: A fully arthroscopic technique. Arthrosc Tech. 2024;13:103035. 10.1016/j.eats.2024.103035 PMC11411331 39308559

[9] Inoue J , Yasui Y , Sasahara J , et al. Comparison of visibility in needle arthroscopy of the ankle according to surgical experience: A cadaveric study. Foot Ankle Surg. 2024;30:603‐607. 10.1016/j.fas.2024.05.005 38762338

[10] Hao KA , Vander Griend RA , Nichols JA , Reb CW . Intraoperative assessment of reduction of the ankle syndesmosis. Curr Rev Musculoskelet Med. 2022;15:344‐352. 10.1007/s12178-022-09769-0 PMC9463424 35829893

[11] Vohra R , Singh A , Thorat B , Patel D . Instability of the distal tibiofibular syndesmosis. J Orthop Surg (Hong Kong). 2023;31:10225536231182349. 10.1177/10225536231182349 37449812

[12] Vinje T , Inderhaug E , Jarning J , et al. Suture‐button repair improves outcomes in arthroscopically diagnosed chronic syndesmotic injury despite low imaging sensitivity. Arthrosc Sports Med Rehabil. 2025;7:101227. 10.1016/j.asmr.2025.101227 PMC12827221 41583811

[13] Andersen MR , Wendt Ræder LB , Stødle AH . Arthroscopic repair of the anterior inferior tibiofibular ligament. Arthrosc Tech. 2025;14:103166. 10.1016/j.eats.2024.103166 PMC11843295 39989685

[14] Wojtowicz BG , Domzalski M , Lesman J . Needle arthroscopic‐assisted repair of tibio‐fibular syndesmosis acute injury: A case report. World J Orthop. 2024;15:820‐827. 10.5312/wjo.v15.i8.820 PMC11331323 39165867

[15] Tham A , Butler JJ , Gianakos AL , Kennedy JG . Needle arthroscopy: Current and future use in the ankle. Foot Ankle Clin. 2025;30:893‐903. 10.1016/j.fcl.2025.07.013 41109735

[16] Degroot H , Al‐Omari AA , El Ghazaly SA . Outcomes of suture button repair of the distal tibiofibular syndesmosis. Foot Ankle Int. 2011;32:250‐256. 10.3113/FAI.2011.0250 21477543

[17] Walinga AB , van der Stappen T , Kerkhoffs GMMJ , Emanuel KS . Only minor complications are reported after needle arthroscopy: A systematic review. Arthrosc Sports Med Rehabil. 2025;7:101158. 10.1016/j.asmr.2025.101158 PMC12447137 40980247

